# Gandouling protects against hepatic fibrosis in Wilson disease through the lncRNA-SNHG7/miR-29b/DNMT3A pathway

**DOI:** 10.1007/s13205-026-04769-0

**Published:** 2026-03-31

**Authors:** Han Wang, Lanting Sun, Xin Yin, Daiping Hua, Qiaoyu Xuan, Jiajia Wang, Wei Dong, Wenming Yang

**Affiliations:** 1 Department of Neurology, The First Affiliated Hospital of Anhui University of Chinese Medicine, 117 Meishan Road, Shushan District, Hefei, 230031 China; 2 Graduate school, Anhui University of Chinese Medicine, Hefei, 230038 China; 3 Department of Ultrasound, The First Affiliated Hospital of Anhui University of Chinese Medicine, Hefei, 230031 China; 4 Key Laboratory of Xin’an Medicine, Ministry of Education, Hefei, 230038 China

**Keywords:** Autophagy, Copper-loaded model, Traditional Chinese medicine, Wilson disease, Gandouling

## Abstract

The therapeutic efficacy of Gandouling (GDL) against hepatic fibrosis, along with its underlying mechanisms, was evaluated in a model of Wilson disease (WD). Using a copper-loaded rat model and in vitro LX-2 cell assays, we comprehensively evaluated the effects of GDL by employing transmission electron microscopy (TEM), histopathology, ultrasound elastography, confocal microscopy (mCherry-GFP-LC3 assay), qRT-PCR, and Western blotting. GDL treatment significantly improved liver function, indicated by reduced serum levels of alanine aminotransferase (ALT), aspartate aminotransferase (AST), hyaluronic acid (HA), laminin (LN), type III pre-collagen (PC-III), and collagen IV (C-IV). GDL also reduced the hepatic copper content and ameliorated histopathological and ultrasonographic signs of fibrosis. At the molecular level, GDL downregulated the expression of lncRNA SNHG7, DNMT3A, α-smooth muscle actin (α-SMA), and collagen I, while upregulating the expression of miR-29b. Furthermore, GDL inhibited autophagy, as shown by reduced levels of Beclin-1 and LC3-II/LC3-I and decreased autophagosome formation. These results demonstrate that GDL alleviates copper overload-induced hepatic fibrosis through modulation of the SNHG7/miR-29b/DNMT3A axis and inhibition of excessive autophagy.

## Introduction

Wilson disease (WD) is an autosomal recessive disorder resulting from mutations in the copper transporter-encoding *ATP7B* gene, causing impaired copper metabolism (Członkowska et al. 2018). In patients with WD, inappropriate copper deposition occurs in many organs, including the brain, kidneys, liver, and cornea. Given the central role of the liver in copper metabolism and its sensitivity to injury, hepatic fibrosis tends to be the first pathological manifestation of WD. Hepatic fibrosis is primarily driven by the myofibroblastic transformation of activated hepatic stellate cells (HSCs), together with progressive extracellular matrix (ECM) accumulation. Elimination of the associated pathogenic factors has been shown to reverse hepatic fibrosis (Seki and Brenner 2015; Sun and Kisseleva 2015), underscoring the importance of developing new antifibrotic interventions.

Autophagy has increasingly been identified as an important factor contributing to the pathogenesis of hepatic fibrosis. When present at excessively high levels, copper is toxic and can activate autophagic activity. Moderate levels of autophagy are essential for the maintenance of cellular homeostasis (Ke 2019; Zischka and Kroemer 2020). However, excessive autophagy can provide an energy source in the form of ATP, which may accelerate fibrotic disease progression by promoting ECM deposition (Cheng et al. 2023). In recent years, noncoding RNAs (ncRNAs) have garnered growing interest as mediators of hepatic fibrosis. Long ncRNAs (lncRNAs) can bind to microRNAs (miRNAs) as competing endogenous RNAs (ceRNAs), thereby regulating the expression of miRNA target genes and altering fibrotic activity (Jiang and Xu 2023). Several lncRNAs have been demonstrated to influence hepatic fibrosis via the autophagic axis (Han et al. 2018; Xie et al. 2019; Zhang et al. 2020). Small nuclear RNA host gene 7 (SNHG7) is a lncRNA expressed in the liver that can regulate autophagic signaling (Chen et al. 2017; Yu et al. 2019). DNA methyltransferase 3 A (DNMT3A) plays an important role in autophagy, and both *DNMT3A* mRNA and the lncRNA *SNHG7* harbor binding sites for miR-29b (Page et al. 2016; Zhang et al. 2018a, b; Hu 2019). The expression of *SNHG7* is inhibited by miR-29b binding, and the pathological downregulation of *DNMT3A* has been found to regulate HSC activation and autophagy, which ultimately affects the onset of liver fibrosis (Xie et al. 2021).

In recent years, considerable progress has been made in the understanding and management of hepatic fibrosis. Gandouling (GDL), which consists of Dahuang (*Radix Et Rhizoma Rhei Palmati*), Ezhu (*Rhizoma Curcumae Phaeocaulis*), Danshen (*Radix Salviae Miltiorrhizae*), Huanglian (*Rhizoma Coptidis*), Jianghuang (*Rhizoma Curcumae Longae*), and Jixueteng (*Caulis Spatholobi*), was first utilized as a therapeutic agent at the First Affiliated Hospital of Anhui University of Chinese Medicine in 2005. The bioactive compounds present in traditional herbs and Chinese medicinal preparations have been demonstrated to exert beneficial effects through the targeting of multiple targets and pathways (Chen et al. 2022). Notably, GDL has been reported to suppress hepatic fibrosis (Cheng et al. 2023), and clinical studies have observed its benefits in patients with WD accompanied by complications such as splenomegaly, hypersplenism, cardiac impairment, and neurological deficits (Ma et al. 2019; Fang et al. 2022; Jiang et al. 2022; Chen et al. 2023). However, the precise mechanisms underlying the anti-fibrotic effects of GDL in WD are not fully understood. In the present study, we hypothesized that GDL mitigates copper overload-induced liver fibrosis by modulating the lncRNA-SNHG7/miR-29b/DNMT3A pathway and regulating autophagy. This was investigated in a copper-loaded rat model, a well-established approach for simulating WD-related copper accumulation and hepatic injury, using male Sprague-Dawley (SD) rats, known for their high tolerance to copper exposure. Penicillamine (PCA), a conventional copper-chelating agent despite its adverse effects with long-term use (Dong and Wu 2021), was included as a positive control. The objective of the study was to provide mechanistic insights into the protective role of GDL against WD-associated hepatic fibrosis.

## Materials and methods

### Animals and treatments

In total, 60 specific pathogen-free (SPF) male SD rats (180–200 g, Hangzhou Ziyuan Laboratory Animal Technology Co.) were housed under controlled conditions in the Animal Center of the School of Pharmacy of the First Affiliated Hospital of Anhui University of Chinese Medicine with unrestricted access to food and water. After one week of acclimatization under standard conditions (ambient temperature of 24 to 26 °C), the rats were randomly allocated to 6 groups (*n* = 10 per group): control, model, PCA, GDL low-dose, GDL medium-dose, and GDL high-dose groups. The model was induced by daily oral gavage of copper sulfate pentahydrate (300 mg/kg) to all groups, except for the control group, for 30 days (Zhang et al. 2023). The control group received an equivalent volume of distilled water daily throughout this period.

Following the successful establishment of the model, drug treatments were administered *via* oral gavage for an additional 30 days. During this period, the copper sulfate gavage was continued for the model, PCA, and GDL groups. The drug treatments were as follows: the GDL low-, medium-, and high-dose groups received GDL (First Affiliated Hospital of Anhui University of Chinese Medicine, Lot No.: Z20050071) at 0.24, 0.48, and 0.96 g/kg, respectively; the PCA group was treated with PCA (Shanghai Medicine Xinyi Pharmaceutical Co, Batch No.: H31022286) at 90 mg/kg; both the control and model groups received equal volumes of distilled water. All dosing volumes were calibrated weekly based on body weight.

**Fig. 1:**
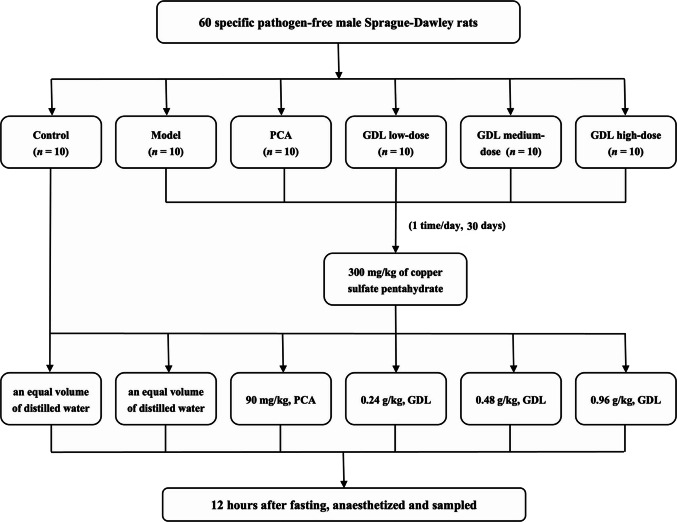
Flowchart of WD copper-loaded rat modeling

After the final administration and following a 12 h fast, 6 rats per group were randomly selected for sample collection. The animals were anesthetized by intraperitoneal injection of 2% sodium pentobarbital (40 mg/kg). Blood samples were collected from the abdominal aorta and were stored at − 20 °C. The liver was rapidly excised; one portion was snap-frozen in liquid nitrogen and stored at − 80 °C for further analysis, while the second portion was fixed in 10% paraformaldehyde for histological examination. The rats were euthanized with an overdose of sodium pentobarbital. All procedures were performed in compliance with institutional ethical guidelines approved by the Experimental Animal Ethics Committee of Anhui University of Chinese Medicine (Approval No.: AHUCM-rats-2023190) (Fig. 1).

### Cell culture and preparation of drug-containing serum

The human HSC cell line LX-2 (Shanghai Saibai Kang, iCell-h128) was purchased from Shanghai Fuhang Biotechnology Co. (Cat No. FH0108). The cells were cultured in DMEM supplemented with 10% FBS and 1% penicillin and streptomycin. Cells were cultured at 37 °C in a humidified atmosphere of 5% CO_2_ and 95% air. Twenty-four male SD rats were randomly divided into the blank and GDL groups, with those in the GDL group receiving 10 times the clinical equivalent of a 70 kg-adult daily dose by gavage. The blank group was given an equivalent amount of saline. Gavage was administered once daily for 7 days. On day 7, blood was collected from the abdominal aorta 12 h after the last dose and was centrifuged at 3000 rpm for 10 min to collect the serum.

### Cell grouping

To identify the optimal concentration and duration of CuSO_4_ treatment, LX-2 cells were divided into 6 groups, and concentrations of 0 (blank control group), 25, 50, 100, 200, and 400 µmol/L CuSO_4_ were added to each group and cultured for 12, 24, 48, and 72 h.

To determine the optimal GDL concentration in the serum and its duration of action, LX-2 cells were also divided into 6 groups, and GDL-containing serum was added to each group at different concentrations (0, 1.25, 2.5, 5, 10, and 20%) and cultured for 12 h, 24 h, 48 h, and 72 h, respectively.

For the formal test, the cells were divided into 3 groups, namely, the control group (LX-2 cells cultured normally without other treatments), the CuSO_4_ group (LX-2 cells treated with the optimal concentration of CuSO_4_ for the optimal length of time), and the CuSO_4_ + GDL group (cells treated as in the CuSO_4_ model group together with the optimal conditions of GDL treatment).

### CCK-8 assays

Cells were collected and resuspended at a density of 5–10 × 10^4^/mL. Cell suspensions (100 µl per well) were seeded into 96-well plates (LabServ). The edge wells of the plates were filled with sterile PBS (Hyclone, USA, SH30256.01) and incubated overnight at 37 °C with 5% CO₂. Varying concentrations of sterilized and filtered CuSO_4_/GDL-containing serum were added to the treatment group in addition to the control group. After incubation for different times, 10 µL of CCK-8 reagent (BIOSS, BA00208) was added to each well, and the incubation was continued for 1 h. The absorbance value of each well was measured at 450 nm in a microplate reader. Blank wells (medium, CCK-8 reagent) were set up at the same time.

### EdU assays

The cells were washed three times with PBS (Cytiva, SH30256.01) and incubated with trypsin in 0.25% EDTA (Biotronik, C0201) at 37℃ for 1–5 min. When the cells had detached from the surface, medium was added to terminate the digestion, and the cells were collected and centrifuged in 15 mL centrifuge tubes at 1000 rpm for 5 min. After the addition of 4 µL of PBS to the wells of a 12-well plate and drying, 40 µL of cell suspension (1 × 10^5^ cells/mL) was placed in each well and incubated until the cells had adhered to the plate surface, after which 500 µL of medium was added and culture was continued. The EdU working solution (Biyun Tian, C0071S) was diluted 1:1000 with medium, preheated to 37 °C, and added to the cells in the plate, followed by incubation for 2 h. After EdU labeling of the cells was completed, the cells were fixed with 4% paraformaldehyde for 20 min, after which they were washed 3 times with PBS-T and incubated with 0.5% Triton X-100 for 30 min, and then washed. DAPI counterstain was added dropwise, incubated at room temperature for 5 min, and washed slowly with PBS-T for 3 min. The slide was sealed with an anti-fluorescence quenching sealer. The fluorescent sections were scanned with a digital section scanner (Panoramic MIDI).

### Histological staining

Samples of liver tissue were collected, fixed, dehydrated, infiltrated, embedded, sectioned at a thickness of approximately 0.5 μm, and stained with hematoxylin and eosin (H&E) or Masson’s trichrome stain (ebiogo, item no.: B006, B022). The sections were examined to assess the cellular arrangement and evidence of inflammation, necrosis, fibrosis, and overall degeneration.

### Liver ultrasound elastography

After the last dose, three rats were randomly selected and fasted for 12 h. Ultrasound elastography was performed by an experienced operator using a Mindray ultrasound diagnostic instrument (Shen Zheng, China). The rats underwent two-dimensional shear-wave elastography (2D-SWE) using a probe with a frequency of 3–12 MHz, and the median value of three measurements per rat was used as the liver stiffness measurement (LSM).

### ELISA

Diluted samples were placed in the wells of a microtiter plate according to the kit instructions, followed by the addition of the HRP coupling reagent. The plate was then sealed with film and incubated at 37 °C for 30 min. After discarding the liquid, the plate was washed five times, and color development was carried out by incubation with the chromogen solution for 10 min at 37 °C in the dark. After stopping the reaction, the optical density (OD) values were read at 450 nm using a microplate reader (RT-6100, Rayto, China). ELISA kits were used to measure the levels of serum aspartate aminotransferase (AST), alanine aminotransferase (ALT) (Nanjing Jianjian Bioengineering Institute, item numbers C010-2-1 and C009-2-1, respectively), hyaluronic acid (HA), laminin (LN), type III pre-collagen (PC-III), collagen IV (C-IV) (Wuhan Genome Science and Technology Co., Ltd, item numbers: JYM0401Ra, JYM0645Ra, JYM0844Ra, and JYM0178Ra, respectively), and hepatic copper, according to the manufacturer’s instructions.

### Measurement of copper content

Hepatic copper contents were measured using a copper assay kit (Complexometric Colorimetry Method, Kit No: E010-1-1, Batch: 20220526, Nanjing Jiancheng Bioengineering Institute) according to the manufacturer’s instructions. Briefly, 100 mg of liver tissue was homogenized in 900 µL of PBS to prepare a 10% (w/v) homogenate, which was then centrifuged at 3000 rpm for 10 min. The resulting supernatant was collected for subsequent analysis. Copper quantification was performed following the kit protocol, with absorbances read with a microplate reader. The protein concentrations in the supernatants were determined using a bicinchoninic acid (BCA) assay kit, as directed. A standard curve was prepared using bovine serum albumin standards, and the BCA working reagent (50:1 ratio) was mixed with the samples and incubated at 37 °C for 30 min. The absorbance at 562 nm was read using a microplate reader. The final copper content was normalized to the total protein concentration and expressed as µmol per gram of protein (µmol/g prot).

### Transmission electron microscopy

For individual animals, a 1 mm^3^ sample of hepatic tissue was fixed using 2.5% glutaraldehyde (TED PELLA INC, Item No. 18426), dehydrated, embedded, and cut with an ultrathin sectioning machine (Leica, No. UC-7) to produce 70 nm sections. These were then stained, and the numbers and structural characteristics of autophagic microsomes were assessed using transmission electron microscopy (TEM) (Nippon Electron, No. JEM1400).

### Quantitative RT-PCR

Liver tissue samples (50–100 mg) and cells were homogenized, and the precipitates were collected. After lysis, the samples were shaken for 5 min and centrifuged (10 min, 1,200 rpm, 4℃), and the supernatants were collected. After adding isopropanol (Shanghai Guangnuo Chemical Technology Co., Ltd., Batch No.: 20240112) and mixing thoroughly, samples were incubated for 30 min and centrifuged (15 min, 12,000 rpm). Total RNA was extracted with TRIzol reagent (Life Technologies, 15596018CN). One microgram of RNA was mixed with 2 µL of 5 × gDNA Eraser Buffer, 1.0 µL of gDNA Eraser (TaKaRa, RR047A), and RNase-free water in a 0.2 mL EP tube, mixed gently, and heated for 2 min at 42℃ on a PCR machine. The 10 µL reaction solution contained 1 µL each of RT Primer Mix and PrimeScript RT Enzyme Mix I, and 4 µL each of RNase Free dH_2_O and 5 × PrimeScript Buffer 2. cDNA was used as a template for fluorescence quantification for reverse transcription. The reaction system contained: 2 × SYBR Green mixture, 5 µL; forward primer (10 µM), 1 µL; reverse primer (10 µM), 1 µL; cDNA, 1 µL; RNase-free water, 2 µL. qRT-PCR was performed with the following settings: 95 °C for 1 min; 40 cycles of 95 °C for 20 s, and 60 °C for 1 min. β-actin and U6 were used as internal references. Relative expression was assessed using the 2^−ΔΔCt^ method. The primers used are shown in Table 1.

**Table 1:** Quantitative RT-PCR primers used in this study

Gene	Forward primer (5’-3’)	Reverse primer (5’-3’)
lncRNA-SNHG7	GTGACTTCGCCTGTGATGGA	GGCCTCTATCTGTACCTTTATTCC
miR-29b	ACACTCCAGCTGGGTTAATGCTAATTGTGAT	TGGTGTCGTGGAGTCG
DNMT3A	ACACTCCAGCTGGGTTAATGCTAATTGTGAT	AGCATTCATTACTGCAATCAC
β-actin	CCCATCTATGAGGGTTACGC	TTTAATGTCACGCACGATTTC
U6	CTCGCTTCGGCAGCACA	AACGCTTCACGAATTTGCGT

### Western blotting

Cell samples (approximately 1 × 10^5^ cells) and liver tissue were collected. The cells and tissues were lysed by adding 600 µl of RIPA buffer (containing 0.6 mM PMSF), centrifuged (15 min, 12,000 rpm, 4 °C), and the supernatants were collected. Proteins were diluted 1:4 in 5X SDS-PAGE sample buffer (Solarbio, Item No. G8200), followed by boiling in a water bath for 15 min. After cooling to room temperature, the samples were separated on SDS-PAGE, transferred to PVDF membranes (Millipore, Item No.IPVH00010), and blocked with 5% skim milk powder. The membranes were then incubated overnight with antibodies against DNMT3A (Bioss, Item No. bs-23029R, 1:2000, Rabbit, 1:1000, Rabbit), p-DNMT3A (Bioss, Item No. bs-14399R, 1:2000, Rabbit), α-SMA (Bioss, Item No. Bsm-33187 M, 1:2000, Mouse; No. bs-0189R, 1:1000, Rabbit), Collagen I (Bioss, Item No. bs-7158R, 1:1000, Rabbit; No. AB260043, 1:1000, Rabbit), Beclin-1 (Abcam, Item No. ab210498, 1:2000, Rabbit; No. ab62472, 1:1000, Rabbit), LC3B (CST, Item No. 3868s, 1:1000, Rabbit; No. 43566 S, 1:1000, Rabbit), and p62 (Bioss, Item No. bs-2951R, 1:1000, Rabbit) at 4℃ with gentle shaking. HRP-labeled secondary antibodies (1:20,000) were then incubated with blots for 1.2 h, followed by three washes with PBST (10 min/wash) and development with an ECL Ultra Sensitive Luminescence Kit (Thermo, Item No. 340958). GAPDH was used as a loading control and densitometric analyses were performed with ImageJ.

### mCherry-EGFP-LC3

Cells in the logarithmic growth phase were digested with trypsin, after which the cells were counted and the concentration adjusted to 2 × 10^5^/mL. The cells were centrifuged, mixed, added 1 mL of cell suspension, and incubated at 37 °C overnight. Before infection, the viral suspensions were thawed slowly on ice. The culture supernatants were removed from the cells, and one-half of the volume of fresh medium was added and mixed gently with the appropriate volume of virus, calculated from the palpated MOI value, for infection. The amount of virus added per well (µL) was calculated as MOI*number of cells/virus titer (PFU/mL) × 1000. A tandem fluorescent mCherry-GFP-LC3 (Hanhen Biology, HBAD-mCherry-EGFP-LC3) adenovirus construct was used to infect LX-2 cells in confocal dishes. The virus-containing medium was aspirated 8–16 h after infection and replaced with fresh complete culture medium to continue the culture. Cells were fixed with anti-fluorescence quenching encapsulant (containing DAPI) (ebiogo, B024) and blocked 36–48 h after infection. Images were collected under a confocal laser scanning microscope (Zeiss, LSM980), and the number of autophagic vesicles (yellow dots) and autophagolysosomes (red dots) in the cells was counted.

### Immunofluorescence

Tissue sections were dried in an oven and then treated with xylene, treated with ethanol, rinsed, subjected to high-pressure antigen repair, washed again, blocked, and probed with appropriate primary and secondary antibodies. The primary antibodies were against Beclin-1 (Abcam, Item No. ab62472) and LC3-II (Bioss, Item No. Bs-2912R). A tissue autofluorescence quencher was then added in a dropwise manner, followed by sealing using an anti-fluorescence quenching sealer and imaging via fluorescent microscopy (3DHISTECH, Hungary).

### Statistical analysis

Data are presented as means ± standard deviation (SD), and were compared using one-way ANOVA with Bonferroni post-hoc correction. Analyses were performed in GraphPad Prism 9.4.1 (San Diego, CA, USA), and *p* < 0.05 was considered statistically significant.

## Results

### Effect of GDL on LX-2 cell viability and proliferation after copper loading

**Fig. 2:**
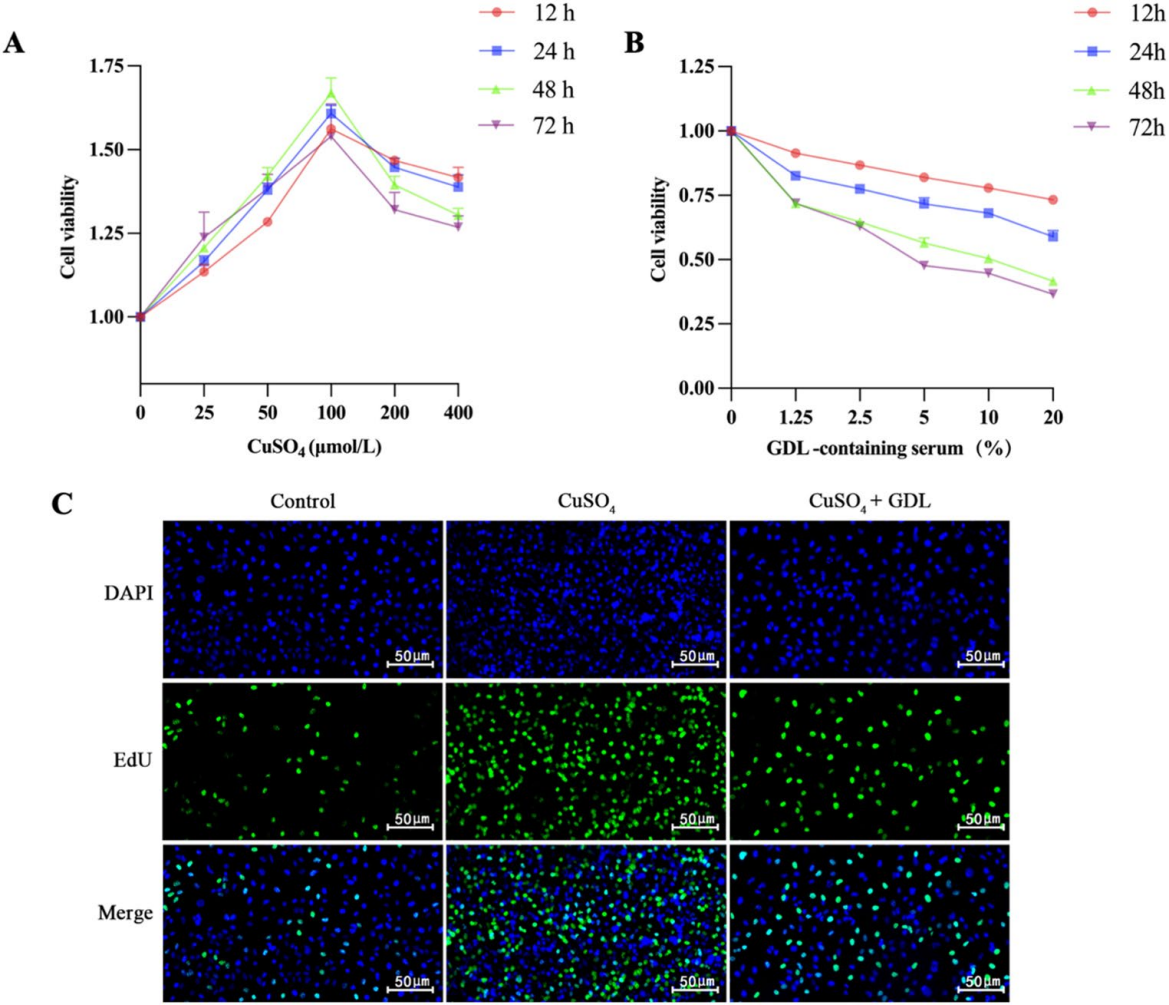
GDL-containing serum reduces copper ion-induced viability in LX-2 cells. **A** Effect of CuSO_4_ on cell viability. **B** Effect of GDL-containing serum on cell viability. **C** Cell proliferation as shown by EdU assays

Compared with the control group, LX-2 cell viability increased significantly after copper ion treatment. The highest cell viability was observed with 100 µmol/L of CuSO_4_. Therefore, 100 µmol/L was selected as the optimal concentration for copper loading. Compared with that at 12 h, the cell viability at 24, 48, and 72 h was significantly increased, with the highest viability observed at 48 h. Therefore, 48 h was selected as the optimal duration of copper loading (Fig. 2A). These optimal conditions were used for treating LX-2 cells, and GDL-containing serum with different concentrations and durations of treatment was used. The results showed that the cell viability was significantly reduced, decreasing with increasing GDL-containing serum concentrations. Based on the potential toxicity and pharmacological properties of the drug, a concentration of 5% GDL-containing serum and a duration of treatment of 48 h were selected as optimal (Fig. 2B). As shown in Fig. 2C, proliferation was higher in cells in the CuSO_4_ group compared with that in the normal group, and the addition of GDL-containing serum reduced the proliferation of the cells.

### GDL treatment improves liver histopathology and stiffness in WD model rats

**Fig. 3:**
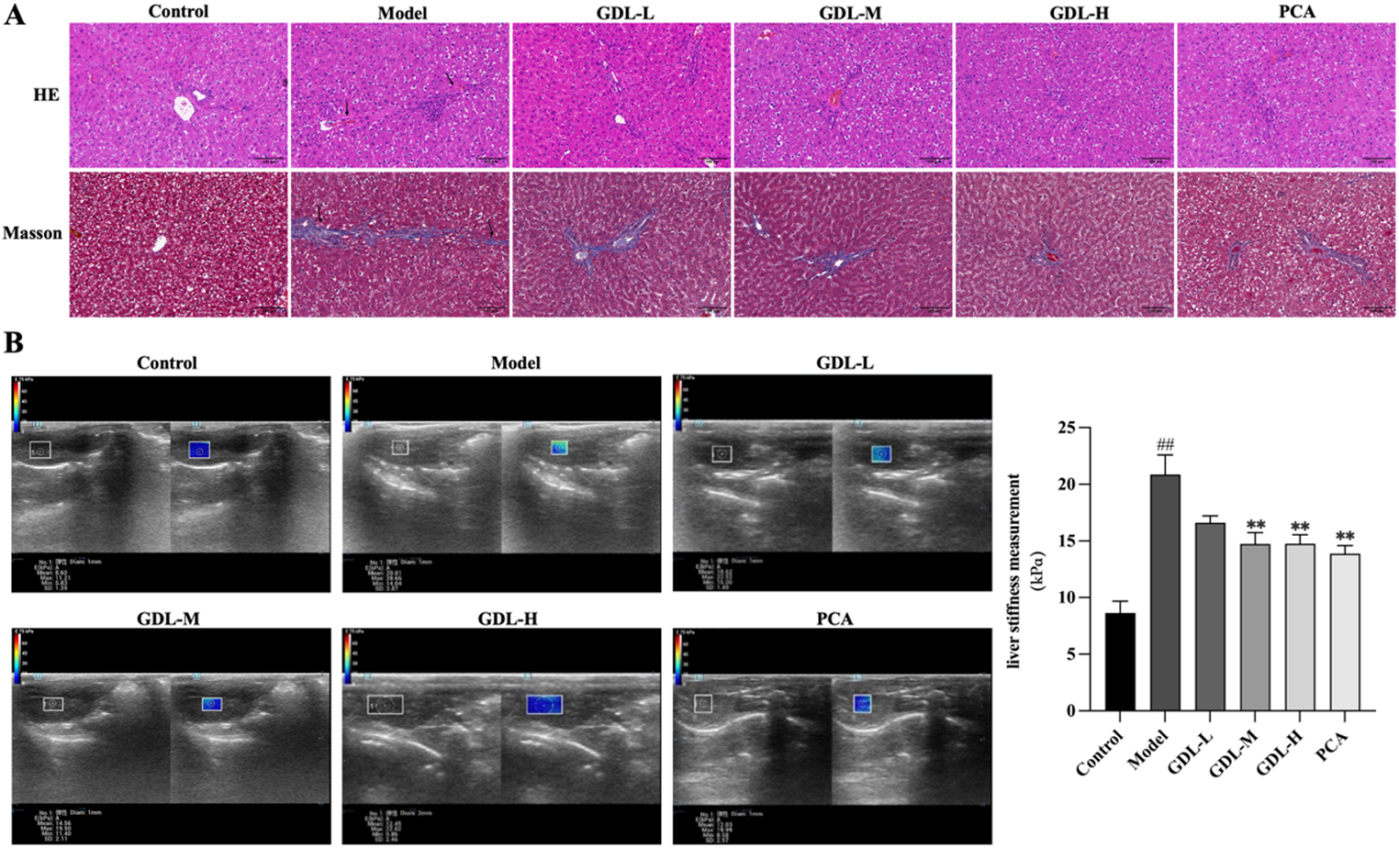
GDL impacts liver histopathology and elasticity in WD model rats. **A** Representative images of liver sections stained with H&E and Masson’s trichrome. (200 × magnification, Scale bar 100 μm). The black arrow points to the area of hepatic fibrosis. **B** The impact of GDL on liver stiffness was analyzed in WD model rats (*n* = 6): Control group (8.60 kPa), Model group (20.81 kPa), GDL-L group (18.62 kPa), GDL-M group (14.56 kPa), GDL-H group (12.45 kPa), PCA group (12.03 kPa). Data are presented as means ± SD. Statistical significance was determined by one-way ANOVA with Bonferroni’s post hoc test. ^##^*p* < 0.01 vs. control group; ^**^p < 0.01 vs. CuSO_4_ group

Liver ultrasound elastrography and histological staining (H&E and Masson’s staining) were used to evaluate liver morphology and elasticity in the experimental rats. These analyses showed that the cells in the control group were tightly arranged, while cells in the model group exhibited some evidence of inflammatory cell infiltration, poorly defined nuclear edges, consolidation, and necrosis. These changes, together with blue collagen fiber deposits detected via Masson’s staining, were all alleviated by GDL and PCA treatment (Fig. 3A). Ultrasonography revealed significantly greater liver stiffness in the model group rats (Fig. 3B), while this stiffness was significantly reduced in the GDL-M, GDL-H, and PCA groups (*n* = 6, *p* < 0.01). These data suggested that WD model rats showed morphological tissue damage in the liver together with abnormal hepatic elasticity, while GDL administration was sufficient to reverse these pathological changes. 

### GDL improves liver function and related serological indicators in WD model rats

**Fig. 4:**
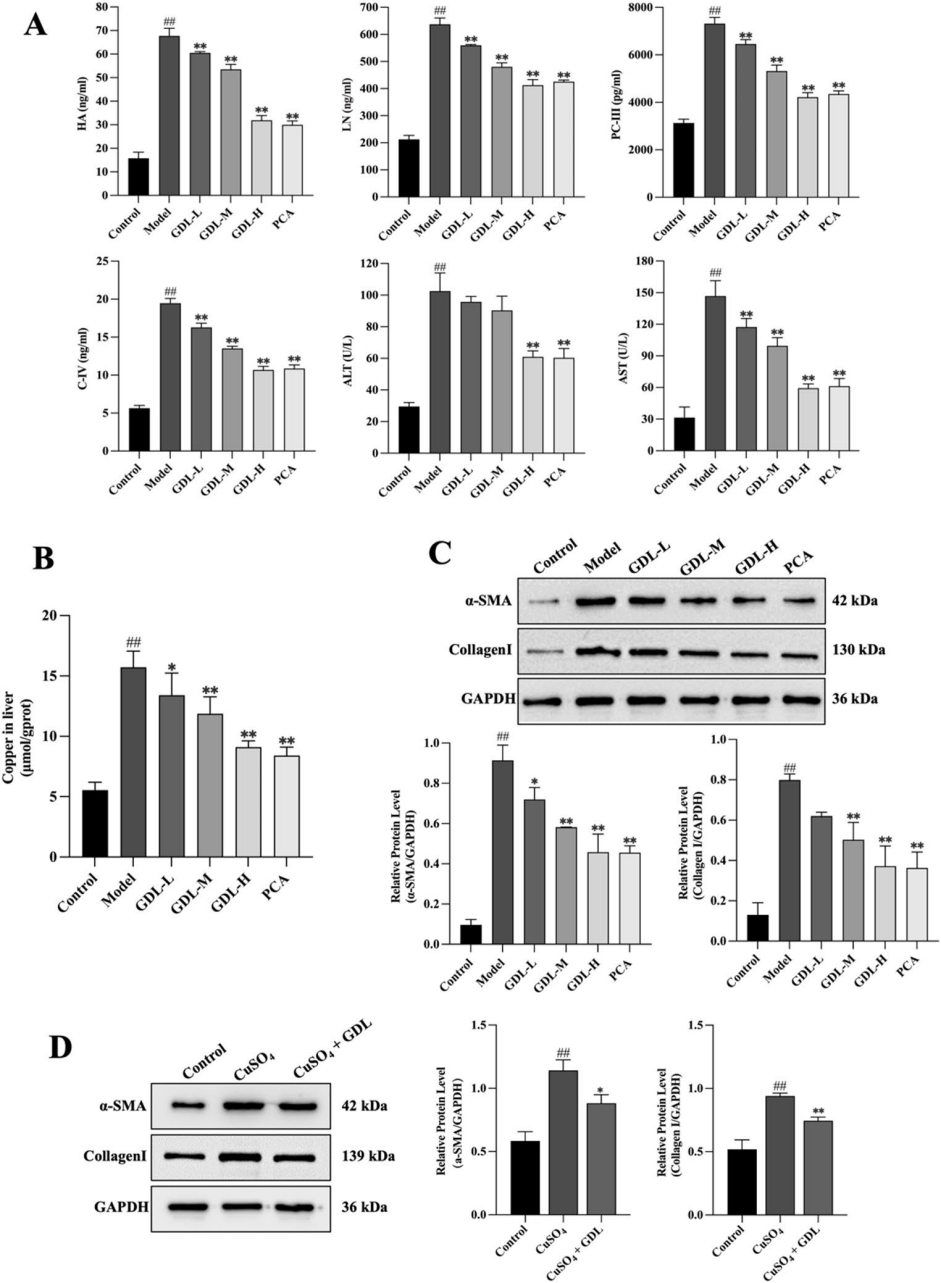
GDL treatment improves liver function and fibrosis-related serological indices in WD model rats. **A** Serum levels of hyaluronic acid (HA), laminin (LN), type III pro-collagen (PC-III), collagen IV (C-IV), aspartate aminotransferase (ALT), and alanine aminotransferase (AST) in each group (*n* = 6). **B** Hepatic copper content across different groups (*n* = 6). **C** Protein expression of α-SMA and collagen I in rat liver tissues, as determined by Western blotting (*n* = 3). **D** Protein expression of α-SMA and collagen I in LX-2 cells treated with CuSO₄ and GDL-containing serum (*n* = 3). GAPDH was used as a loading control. Data are presented as means ± SD. Statistical significance was determined by one-way ANOVA with Bonferroni’s post hoc test. ^##^*p* < 0.01 vs. control group; ^*^p < 0.05, ^**^p < 0.01 vs. model group; ^*^p < 0.05, ^**^p < 0.01 vs. CuSO_4_ group

As shown in Fig. 4A, relative to the control group, the model group rats showed increased HA, LN, PC-III, and C-IV levels (*p* < 0.01), higher AST and ALT levels (*p* < 0.01), and an increase in hepatic copper content (*p* < 0.01). HA, LN, PC-III, and C-IV levels were decreased in the GDL-M, GDL-H, and PCA groups compared to the model group (*p* < 0.01). Reduced AST and ALT levels were observed in the GDL-H and PCA groups (*p* < 0.01). (Fig. 4B) Both GDL and PCA reduced hepatic copper levels (GDL-L: *p* < 0.05; GDL-M, GDL-H, and PCA: *p* < 0.01). Western blotting was used to assess indices related to HSC activation (Fig. 4C), which revealed increased α-SMA and collagen I levels in the model group rats (*p* < 0.01). Lower α-SMA expression was evident following treatment in the GDL and PCA groups (GDL-L: *p* < 0.05, GDL-M, GDL-H, GDL-H, PCA: *p* < 0.01), while reduced collagen I levels were apparent in the GDL-M, GDL-H, and PCA groups (*p* < 0.01). Fig. 4D shows the effect of GDL on α-SMA and collagen I protein expression in LX-2 cells induced by copper loading, suggesting that α-SMA and collagen I protein expression was elevated in the CuSO_4_ group. GDL-containing serum reduced the expression levels of α-SMA and collagen I proteins compared with the model group. These data demonstrated the ability of GDL to suppress HSC activation, thus preserving liver integrity and functionality and thereby protecting against hepatic fibrosis. 

### GDL treatment alters hepatic ultrastructural characteristics in WD model rats

**Fig. 5:**
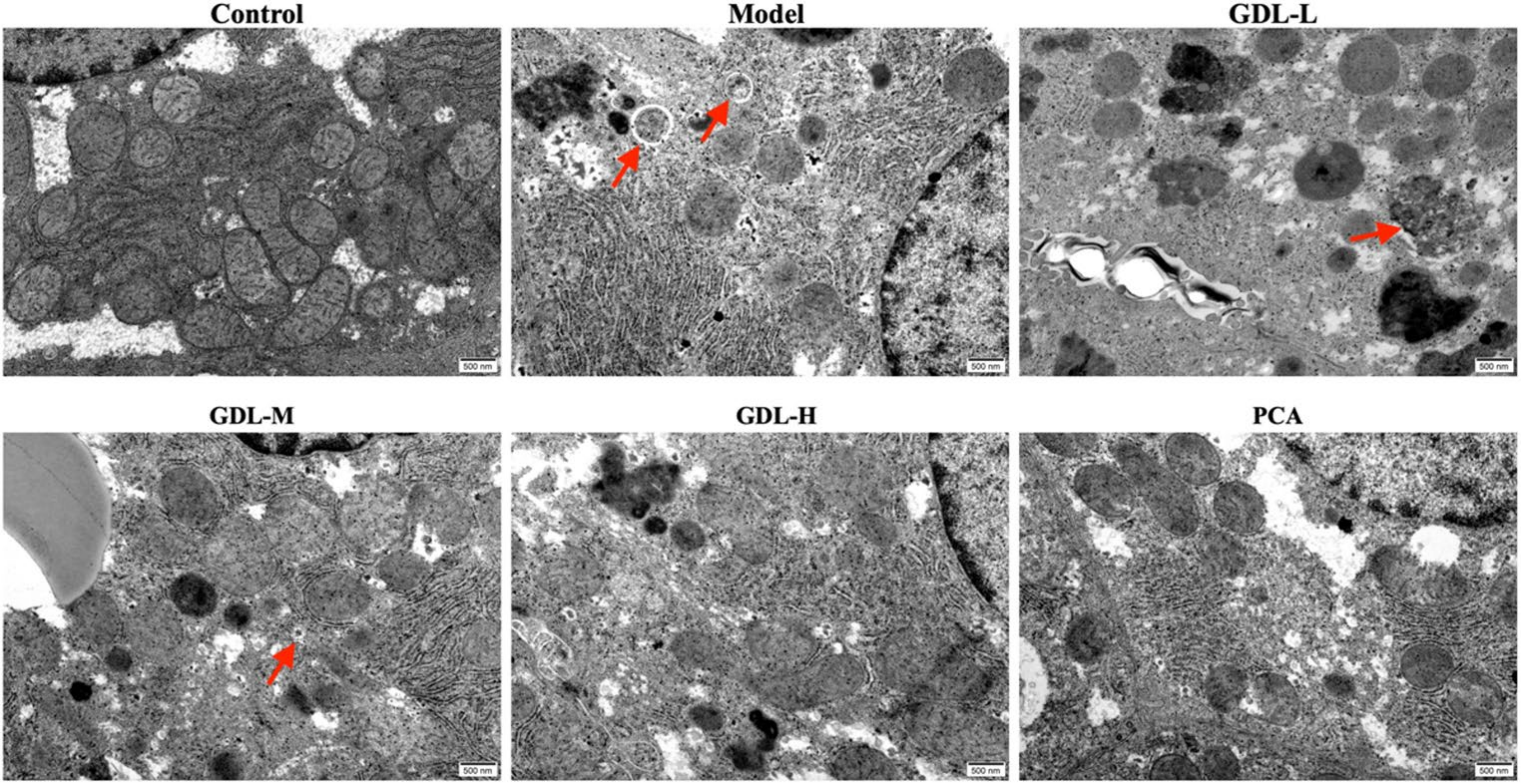
TEM images showing ultrastructural changes in hepatocytes from each group (Original magnification: 25000; scale bar: 500 nm). Note: Autophagosomes are denoted by red arrows

TEM analysis showed that the cellular structures in the control group were distinct, with abundant mitochondria, endoplasmic reticulum (ER), and Golgi structures and no apparent autophagosomes (Fig. 5). However, in the model group, poorly defined cell structures, ER swelling and breakage, mitochondrial vacuolization, and autophagosome formation were all apparent, with several autophagolysosomes being evident, suggesting the activation of protective mechanisms following cellular injury. Reduced autophagosome numbers and improved structural characteristics were observed in the GDL and PCA treatment groups, with comparable efficacy between the GDL-H and PCA groups. 

### GDL suppresses autophagic activity

#### Autophagy in liver tissue

**Fig. 6:**
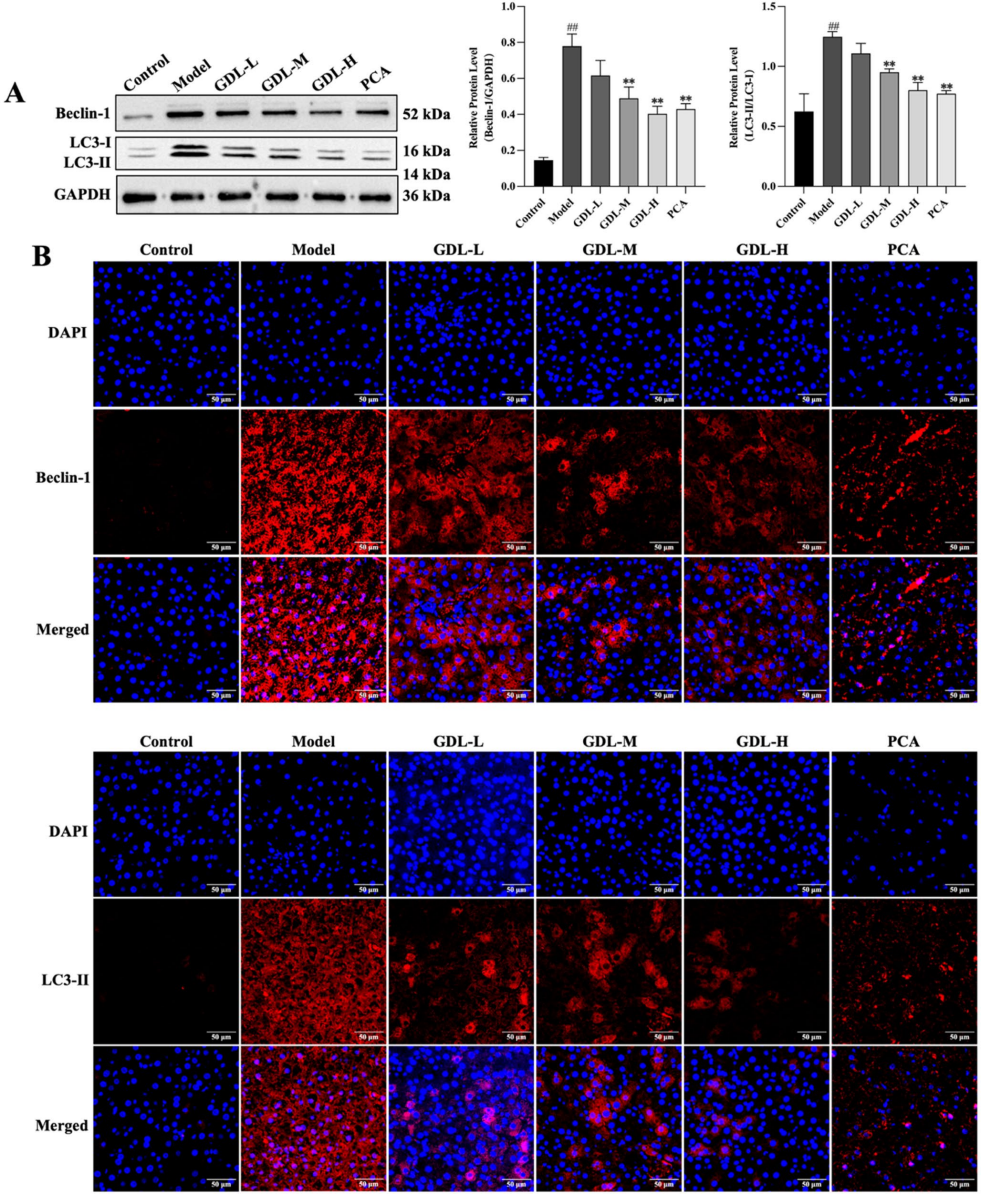
GDL inhibits autophagic activity in liver tissue. **A** Autophagy-related protein expression was analyzed in hepatocytes from the indicated treatment groups by Western blotting (*n* = 3). GAPDH was used as a loading control. Data are presented as means ± SD. Statistical significance was determined by one-way ANOVA with Bonferroni’s post hoc test. ^##^*p* < 0.01 vs. control group; ^**^p < 0.01 vs. model group. **B** Beclin-1 and LC3-II were detected through immunofluorescent staining. Nuclei were counterstained with DAPI (Original magnification: 400; scale bar: 50 μm)

The levels of the autophagy markers Beclin-1 and LC3-II were next analyzed using immunofluorescent staining and Western blotting. (Fig. 6A) Western blotting demonstrated significantly increased Beclin-1 and LC3-II/LC3-I levels in the model group (*p* < 0.01), while these levels were significantly reduced in the GDL-M, GDL-H, and PCA groups following treatment (GDL-M, GDL-H, and PCA: *p* < 0.01). Immunofluorescent staining consistently revealed increased Beclin-1 and LC3-II signal intensities in the model group, while GDL treatment reversed these increases (Fig. 6B).

#### Autophagy in LX-2 cells

**Fig. 7:**
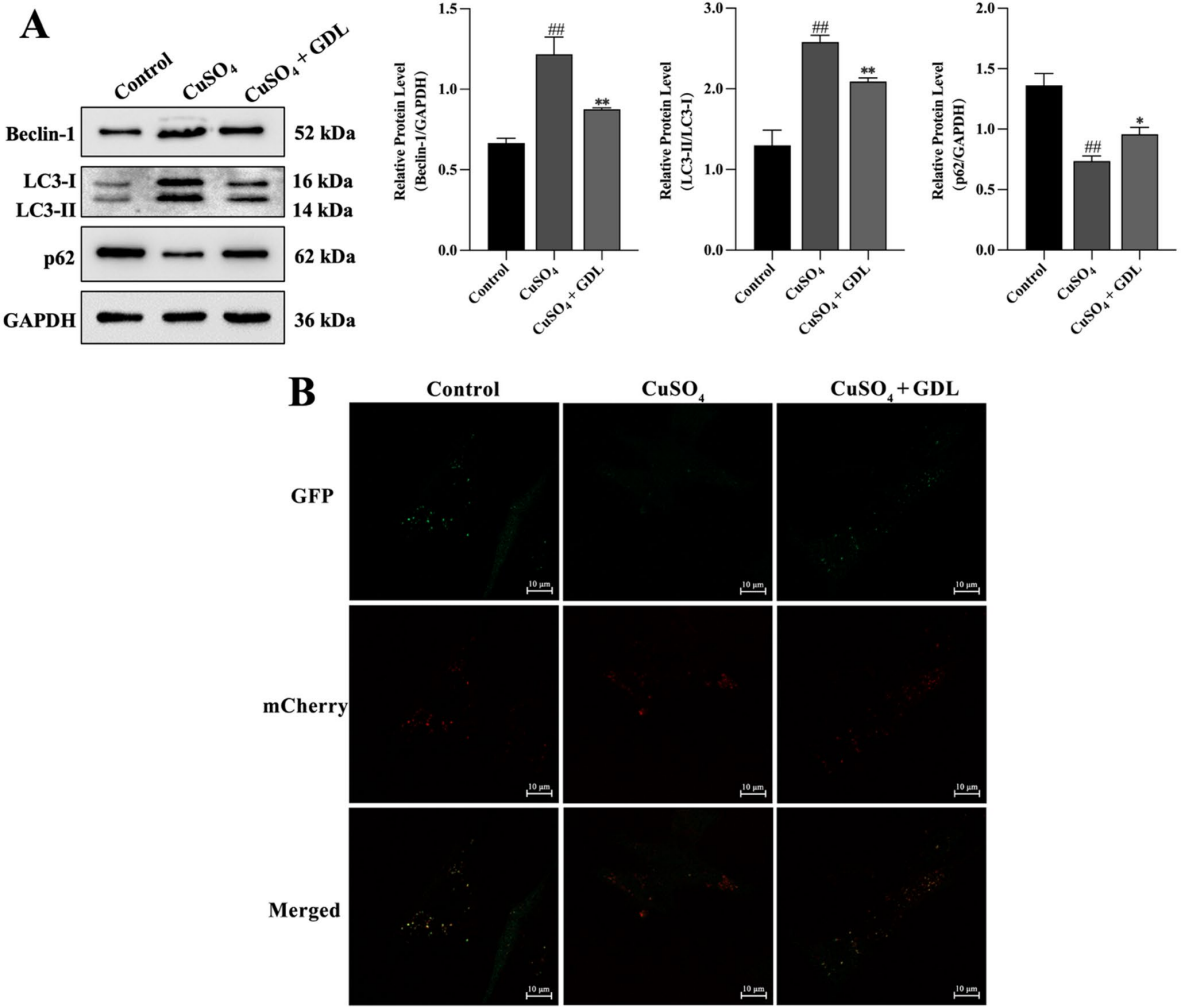
GDL inhibits autophagic activity in LX-2 cells. **A** Western blotting analysis of Beclin-1, LC3-II/LC3-I, and p62 expression (*n* = 3). GAPDH was used as a loading control. Data are presented as means ± SD. ^##^*p* < 0.01 vs. control group; ^*^p < 0.05, ^**^p < 0.01 vs. CuSO_4_ group. **B** Representative fluorescent images of cells following mCherry-GFP-LC3 adenovirus infection (Scale bar: 10 μm). In the merged image, yellow spots indicate autophagosomes, while red spots indicate autophagic lysosomes. The degree of autophagic flux can be seen by the number of different color spots

The findings suggested that GDL attenuated autophagy levels in liver fibrosis. For further verification, the protein levels of Beclin-1, LC3-II/LC3-I, and p62 were examined in LX-2 cells using Western blotting. LX-2 cells were transfected with the mRFP-GFP-LC3 adenovirus construct to assess the number of autophagosomes and autolysosomes in *vitro*. Fig. 7A shows that Beclin-1 and LC3-II/LC3-I expression was elevated in the CuSO_4_ group (Beclin-1, LC3-II/LC3-I: *p* < 0.01), while the protein expression of p62 was reduced (*p* < 0.05). This trend was reversed following the addition of GDL-containing serum. Beclin-1 and LC3-II/LC3-I protein levels were reduced in the CuSO_4_ + GDL group (Beclin-1, LC3-II/LC3-I: *p* < 0.01), while the levels of p62 were increased (*p* < 0.05) (Fig. 7B). The number of autophagosomes was then assessed in vitro, showing that compared with the control group, autophagosome numbers were increased in the CuSO_4_ group. After the addition of GDL-containing serum, the number of autophagosomes was decreased in the CuSO_4_ + GDL group.

#### GDL targets the lncRNA-SNHG7/miR-29b/DNMT3A axis

**Fig. 8:**
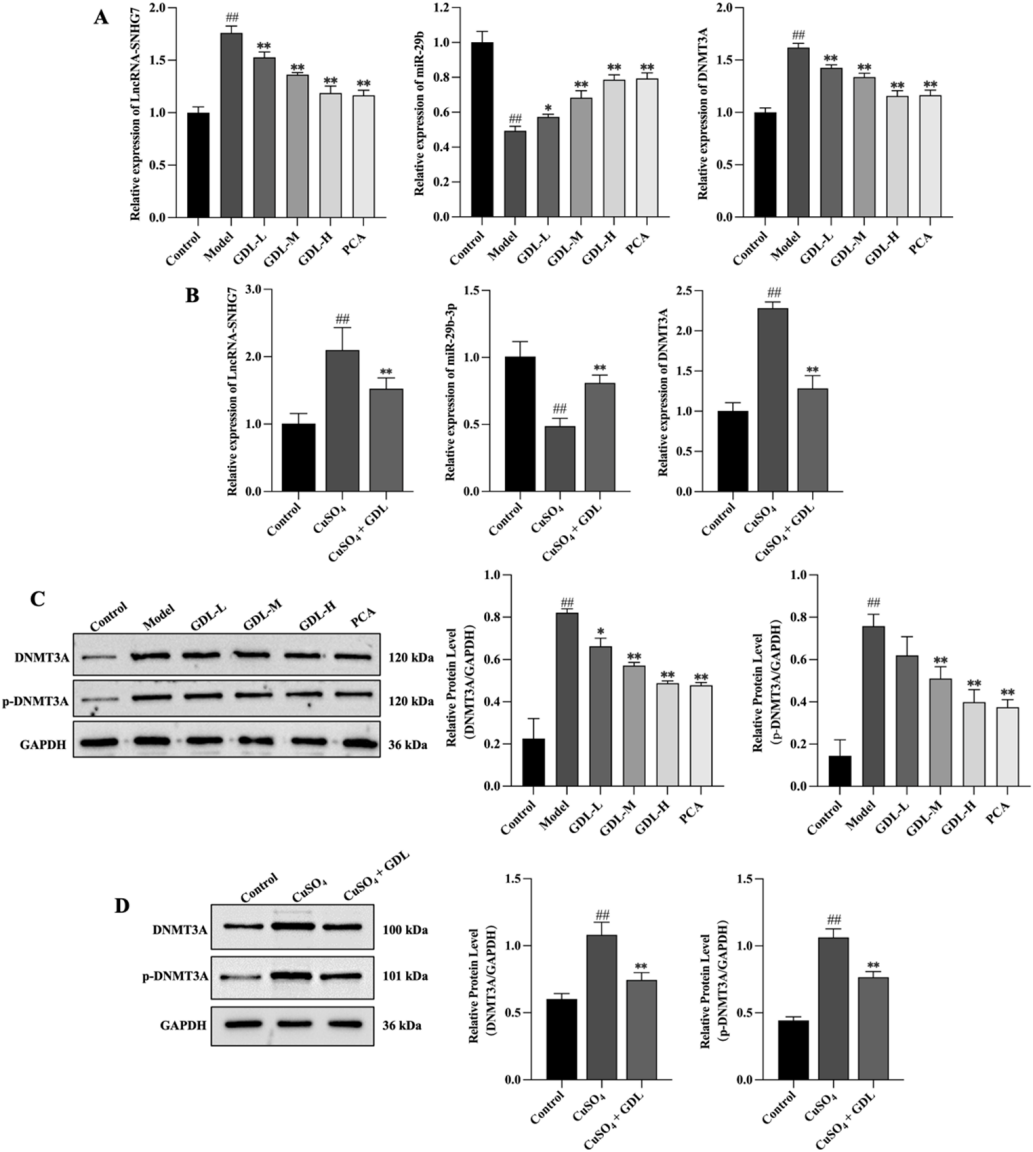
GDL regulates the SNHG7/miR-29b/DNMT3A axis in *vivo* and in *vitro*. **A** Expression of SNHG7, miR-29b, and DNMT3A in liver tissues (*n* = 6). **B** Expression of SNHG7, miR-29b, and DNMT3A in LX-2 cells (*n* = 6). **C** Protein levels of DNMT3A and p-DNMT3A in liver tissues (*n* = 3). **D** Protein levels of DNMT3A and p-DNMT3A in LX-2 cells (*n* = 3). GAPDH was used as a loading control. Data are presented as means ± SD. ^##^*p* < 0.01 vs. control group; ^*^p < 0.05, ^**^p < 0.01 vs. model group; ^**^p < 0.01 vs. CuSO_4_ group

To understand the effects of GDL on the lncRNA-SNHG7/miR-29b/DNMT3A axis, we examined the expression of SNHG7, miR-29b, and DNMT3A in rat liver tissue samples and LX-2 cells using qRT-PCR. In addition, the protein expression of DNMT3A and p-DNMT3A in liver tissues and LX-2 cells was evaluated using Western blotting. Fig. 8A shows significant increases in SNHG7 and DNMT3A expression in the model group, together with reduced miR-29b expression (*p* < 0.01). Significant reductions in SNHG7 and DNMT3A expression were observed in all GDL and PCA treatment groups (*p* < 0.01), while miR-29b expression was increased (GDL-L: *p* < 0.05; GDL-M, GDL-H, and PCA: *p* < 0.01) (Fig. 8B). Similarly, the levels of SNHG7, miR-29b, and DNMT3A in LX-2 cells were consistent with the above results. Treatment with GDL-containing serum, however, reduced the expression of both SNHG7 and DNMT3A and that of miR-29b (*p* < 0.01). Consistently, as shown in Fig. 8C, Western blotting revealed significant increases in DNMT3A and p-DNMT3A levels in rats from the model group (*p* < 0.01), while GDL and PCA treatment led to significant reductions in DNMT3A (GDL-L: *p* < 0.05, GDL-M: *p* < 0.01, GDL-H: *p* < 0.01, PCA: *p* < 0.01) and p-DNMT3A (GDL-M, GDL-H, and PCA: *p* < 0.01) levels. The results shown in Fig. 8D verify these results. Both DNMT3A and P-DNMT3A were highly expressed in the CuSO_4_ group (*p* < 0.01), while this trend was reversed in the CuSO_4_ + GDL group (*p* < 0.01).

## Discussion

The present study demonstrates that GDL was effective in alleviating copper overload-induced hepatic fibrosis, primarily through modulation of the SNHG7/miR-29b/DNMT3A signaling axis and the suppression of autophagy. Using integrated in vivo and in vitro models of WD, it was observed that GDL treatment significantly reduced copper accumulation in the liver, improved liver function, and ameliorated histological and biochemical markers of fibrosis.

GDL is a well-established treatment for WD, exhibiting activities such as clearing heat and removing toxins, eliminating phlegm, and transforming stasis. *Rhizoma Curcumae Longae*, a principal component of GDL, has been shown to promote copper excretion (Zhang et al. 2018a, b; Sun et al. 2023), consistent with the present findings that GDL reduced liver copper content and improved serum parameters of liver function. Additionally, GDL downregulated the expression of α-SMA and collagen I, supporting its ability to inhibit hepatic stellate cell activation and extracellular matrix deposition (Cheng et al. 2023).

Copper deposition is known to induce autophagy (Zischka et al. 2011), a finding consistent with our present results. Notably, GDL treatment was effective in suppressing copper-mediated autophagy. Previous studies have also reported upregulation of autophagy in WD model mice, where it contributes to HSC activation and excessive ECM accumulation (Li et al. 2018; Xiu et al. 2021), ultimately promoting fibrogenesis. Autophagy is widely recognized as an energy supply mechanism that supports HSC activation during fibrotic progression (Hernández-Gea and Friedman 2012; Hernández-Gea et al. 2012). These findings highlight the close link between autophagy and hepatic fibrosis, suggesting that during fibrotic disease progression, autophagic activity transitions from a protective to a harmful role. In the present CuSO_4_-induced WD rat model, higher levels of autophagic activity were evident, whereas they were reduced with GDL and PCA treatment, with this effect being particularly pronounced in the GDL-H group. In addition, the LX-2 cell results showed that autophagy levels were elevated in the liver fibrosis CuSO_4_ group, and that GDL-containing serum reduced the autophagy levels. These results suggest that modulation of autophagy represents a key mechanism through which GDL restores liver function and alleviates hepatic fibrosis.

lncRNAs, which are frequently dysregulated in human diseases such as cancer, have been implicated in a broad spectrum of biological processes, including chromatin remodeling, transcriptional control, and post-transcriptional modulation (Peng et al. 2018). In terms of post-transcriptional regulation, lncRNAs can serve as ceRNAs to sponge miRNAs, leading to the derepression of miRNA targets (Yu et al. 2020). For instance, HOTAIR was shown to inhibit PTEN expression by sponging miR-29b (Yu et al. 2020). Similarly, SNHG7 promotes the progression and liver metastasis of colorectal cancer by sponging miR-216b (Shan et al. 2018). Elevated SNHG7 expression has also been observed in hepatic fibrosis (Wu et al. 2020), and its knockdown can reduce collagen deposition and the levels of fibrotic proteins, indicating suppression of HSC activation (Yu et al. 2017; Nielsen et al. 2019), supporting a pro-fibrotic role for SNHG7. On the other hand, DNMT3A, a DNA methyltransferase, induces HSC activation and contributes to fibrogenesis (Garzon et al. 2009; Page et al. 2016). DNMT3A-mediated methylation promotes hepatic fibrosis, while inhibition of methylation has protective effects in models of biliary atresia (Wang et al. 2017,Wang et al. 2019). Xie et al. (Xie et al. 2021) further identified a miR-29b binding site in SNHG7 and demonstrated that modulation of SNHG7 or miR-29b influences DNMT3A expression. Moreover, SNHG7 knockdown in activated HSCs was found to downregulate α-SMA, collagen I, LC3-II/LC3-I, and Beclin-1, accompanied by autophagy inhibition, suggesting that the SNHG7/miR-29b/DNMT3A axis modulates hepatic fibrosis partly through regulating autophagy. Our findings showed increased expression of SNHG7 and DNMT3A, along with reduced miR-29b levels, in fibrotic liver tissues. GDL treatment was effective in reversing these changes, demonstrating its regulatory role in the SNHG7/miR-29b/DNMT3A pathway. Previous studies have indicated that GDL ameliorates liver fibrosis by modulating autophagy, and the present results further suggest that the anti-autophagic activity of GDL is mediated, at least in part, through suppression of this signaling axis, ultimately attenuating the progression of fibrosis.

The findings of this study have important clinical implications for the treatment of WD. Current pharmacological management of WD relies primarily on copper-chelating agents (such as PCA and trientine), which promote copper mobilization and urinary excretion, and on zinc salts, which inhibit intestinal absorption of copper (Ferenci 2004; Roberts and Schilsky 2008). Although these treatments are effective in reducing copper overload and preventing disease progression, several important limitations remain. Notably, a significant proportion of patients with neurological manifestations experience worsening of their condition following the initiation of chelation therapy (Kumar et al. 2022; Mohr et al. 2023). In contrast, GDL exhibits a multi-targeted mechanism that includes copper elimination, anti-fibrotic activity, and autophagy modulation.

Several limitations of this study should be acknowledged. First, while the copper sulfate-induced model is effective in mimicking copper overload and liver fibrosis, both hallmarks of WD, it is an induced model and does not fully represent the genetic etiology or chronic progression of human WD. Therefore, the interpretation of our results focuses primarily on the anti-fibrotic and autophagy-modulating effects of GDL under conditions of copper-induced injury. Second, although the present data suggest the involvement of the SNHG7/miR-29b/DNMT3A axis in the effects of GDL, direct mechanistic verification through gain- and loss-of-function experiments (e.g., SNHG7 knockdown, miR-29b mimic/inhibitor rescue studies) is lacking. Thus, while our findings support an association between GDL and this pathway, future studies are needed to definitively establish causality. Finally, future research utilizing genetic animal models of WD (e.g., *ATP7B*^−/−^ mice) would be valuable in confirming the therapeutic effects and mechanisms of GDL.

## Conclusion

In conclusion, the study findings demonstrated the ability of GDL to protect against copper overload-associated hepatic fibrosis. The underlying mechanisms appear to involve the suppression of autophagic activity through modulation of the lncRNA-SNHG7/miR-29b/DNMT3A axis. This study thus offers a foundation for future studies on the hepatoprotective benefits of GDL as a means of combating or preventing hepatic fibrosis.

## Data Availability

All data generated or analyzed during this study are included in the article/Supplementary Material, further inquiries can be directed to the author.
